# Revision Rates and Associated Risk Factors after Shoulder Arthroplasty

**DOI:** 10.3390/jcm11247256

**Published:** 2022-12-07

**Authors:** Nike Walter, David W. Lowenberg, Steven M. Kurtz, Volker Alt, Edmund C. Lau, Markus Rupp

**Affiliations:** 1 Department of Trauma Surgery, University Hospital Regensburg, 93053 Regensburg, Germany; 2 Department of Psychosomatic Medicine, University Hospital Regensburg, 93053 Regensburg, Germany; 3 Department of Orthopaedic Surgery, Stanford University School of Medicine, Stanford, CA 94063, USA; 4 Implant Research Center, Drexel University, Philadelphia, PA 19104, USA; 5 Exponent Inc., Palo Alto, CA 94025, USA

**Keywords:** proximal humerus fracture, shoulder arthroplasty, revision risk factors, osteoarthritis, rotator cuff injury

## Abstract

This study aims at answering the following questions (1) How high is the revision rate after osteoarthritis-, and rotator cuff-related compared to proximal humerus fracture (PHF)-related shoulder arthroplasty? (2) What are the associated risk factors for a revision after shoulder arthroplasty? Shoulder arthroplasty procedures occurring between 1 January 2009 and 31 December 2019 were identified from the Medicare database. First, revision rates for PHF patients and age- and sex-matched non-fracture patients, grouped into osteoarthritis-related and rotator cuff-related arthroplasty, were compared. Second, revision rates between total shoulder arthroplasty and hemiarthroplasty after PHF were compared. Semiparametric Cox regression was applied, incorporating 23 demographic, clinical, and socioeconomic covariates, to investigate risk factors for revision surgery. Between the considered time period from 2009 through 2019, a total number of 47,979 PHFs was identified. A shoulder arthroplasty procedure was performed in n = 2639 (5.5%, 95%CI: 4.8–6.1) of the cases. The five-year survivorship of the implant was 96.3 (95%CI: 93.8–97.9) after hemiarthroplasty and 96.1% (05%CI: 94.2–97.3) after total shoulder arthroplasty. To compare the revision rates, n = 14,775 patients with osteoarthritis and n = 4268 patients with rotator cuff arthropathy, who received a shoulder arthroplasty, served as a non-fracture control group. Patients receiving a rotator cuff-related arthroplasty were more likely to require a revision compared to patients treated for osteoarthritis (HR: 1.27, 95%CI: 1.04–1.44, *p* = 0.018). Identified significant risk factors for revision surgery after shoulder arthroplasty included age ≤ 75 years, male sex, and osteoporosis. High implant survival was found for hemiarthroplasty and total shoulder arthroplasty for the treatment of PHF in elderly patients. The risk of revision surgery was elevated in patients receiving a rotator cuff-related arthroplasty as well as in patients with osteoporosis, male patients and patients older than 75 years.

## 1. Introduction

Over the last decades, shoulder arthroplasty procedures have drastically increased in the U.S. [1,2,3]. Based on data from the National Inpatient Sample (NIS), it was estimated that 823,361 patients (95%CI: 809,267 to 837,129 patients) were living with a shoulder replacement in 2017 [4]. In addition, numbers were projected to be a 235% increase, with 350,558 procedures by 2025 [1]. One possible reason represents the expanding use and indications. For instance, shoulder arthroplasty procedures are favored in the treatment of primary glenohumeral osteoarthritis and are frequently used for the management of cuff tear arthropathy [5,6,7]. In addition, for proximal humerus fractures (PHF), representing the third most prevalent osteoporotic fracture type, shoulder arthroplasty seems to offer a treatment option with reported satisfactory functional outcomes, good range of motion and pain relief, particularly in elderly patients with three- and four-part PHF [8,9,10,11]. Noteworthy, a consensus on the optimal treatment of PHF is still missing. However, while the majority of PHF is managed conservatively, in recent years, an increasing trend in total shoulder arthroplasty (TSA) has been reported [12,13]. In 2019, patients were significantly more likely to receive a reverse total shoulder arthroplasty (RSA) (OR 22.65) compared to TSA, open reduction and internal fixation, closed reduction and percutaneous pinning, hemiarthroplasty, and intramedullary nailing in 2010 [13]. Importantly, with the increasing performance of shoulder arthroplasty procedures, also the risk of complications such as periprosthetic joint infection is heightened. A few studies reported revision rates based on large registry data such as the Nordic Arthroplasty Register Association [14,15,16], the Swedish Fracture Register [17] and the Australian Orthopaedic Association National Joint Replacement Registry [18]. However, substantial heterogeneity among studies reporting reoperation rates was noted [19]. A recent study from the UK reported that lifetime risks of revision surgery ranged from 1 in 37 (2.7%, 95% CI 2.6% to 2.8%) in women aged 85 years and older to 1 in 4 (23.6%, 23.2% to 24.0%) in men aged 55–59 years. [20]. In addition, an approximately 392% increase in the incidence of revision shoulder arthroplasty from 2216 procedures to 10,290 procedures between 2002 through 2017 was reported, whereby cumulative costs for revision procedures increased from $26 million to $206 million [4].

Thus, given the clinical importance, this study aimed at answering the following questions: (1) How high is the revision rate after osteoarthritis-, and rotator cuff-related compared to proximal humerus fracture (PHF)-related shoulder arthroplasty? (2) What are the associated risk factors for a revision after shoulder arthroplasty?

## 2. Materials and Methods

This study is based on data from Medicare physician service records. These records encompassed diagnoses and treatments rendered in medical offices, outpatient clinics, hospitals, emergency departments, skilled nursing homes, and other healthcare facilities. These records were compiled by the Centers for Medicare and Medicaid Services (CMS) and, after deidentification, were made available for research, known as the Limited Data Set (LDS). Specifically, physician records associated with a 5% sample of Medicare beneficiaries, equivalent to the records from approximately 2.5 million enrollees, formed the basis of this study. The 5% extracts are randomly retrieved from the 100% files. CMS replaced the beneficiary’s identity with a synthetic and unique ID in the LDS data sets, which allowed patients to be followed longitudinally for survivorship and outcomes analyses. Because these LDS datasets were generated from the Medicare fee-for-service enrollees, those enrolled in a Medicare health maintenance organization (HMO), those younger than 65, and those residing outside of the 50 US states were excluded. Since the CMS data is deidentified, it was exempt from review by the Institutional Review Board.

The International Classification of Diseases, Ninth and Tenth Revisions, were used to identify proximal humerus fractures occurring between 1 January 2009 and 31 December 2019 from these physician records. Diagnosis in claims submitted before 1 October 2015 was recorded in ICD-9-CM and thereinafter in ICD-10-CM. The ICD-9 codes used were 812.0 and 812.1 and the ICD-10 codes used were S42.2xxA and S42.2xxB. Treatments were identified by a set of Common Procedural Terminology (CPT) codes as used in previous studies [21]. First, we compared revision rates for humerus fracture patients and other age- and sex-matched non-fracture patients. For non-fracture patients, they were grouped into osteoarthritis-related and rotator cuff-related arthroplasty using the definition in Floyd (2020) [22]. Floyd’s study also separately called out rheumatoid arthritis patients, but the number of cases from this study data were too small to provide reliable calculations and was not included. Second, we compared revision rates between total shoulder arthroplasty and hemiarthroplasty after proximal humerus fracture. The failure rate or the rate of revision after shoulder arthroplasty was investigated using survival analysis techniques. Here, the Kaplan-Meier (KM) method with the Fine and Gray sub-distribution adaptation to account for competing risk was used. Then we used the semiparametric Cox regression to compare revision risk among patient groups. The Cox models incorporated demographic, clinical, and several community-level social-economic measures as covariates. The demographic factors included: age, sex, race, resident region, and Medicare buy-in (as a surrogate for the patient’s economic status). Clinical factors included were osteoporosis, obesity, diabetes mellitus, rheumatoid disease, chronic kidney disease, tobacco dependence, regular use of anti-coagulant, hypertensive disease, ischemic heart disease, cerebrovascular disease, chronic obstructive pulmonary disease (COPD), and congestive heart failure. These conditions were identified from physician records in a one-year period prior to the fracture. These conditions could appear as either primary or secondary diagnoses, but at least two mentions of such conditions in the prior year were required. Table A1 in Appendix A documented the codes used to identify these conditions. The socioeconomic measures for the patients’ resident county included population, median household income, percent of the population with at least college education, percent of the population in poverty, percent of unemployment, and a measure of the urban-rural character of the county. These measures were obtained from the US Census, the Census Bureau’s American Community Survey (ACS) program (Data can be accessed at: https://www.census.gov/programs-surveys/acs/data.html, last accessed: 3 October 2022), and the Economic Research Service of the USDA (data can be accessed at: https://www.ers.usda.gov/data-products/county-level-data-sets/, last accessed: 3 October 2022).

All data processing and statistical analyses were performed using the SAS statistical software (Version 9.4, Cary, NC, USA), and significance was determined at α = 0.05.

## 3. Results

**Figure 1:**
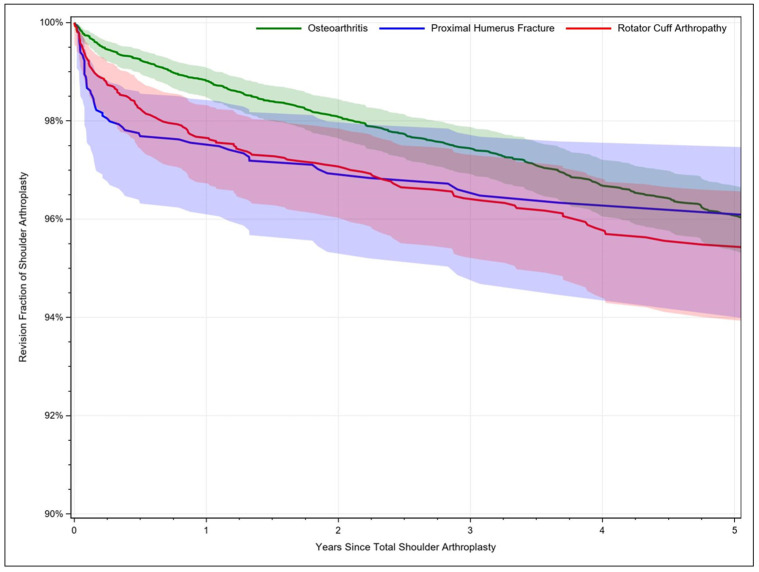
Comparison of revision rates after total shoulder arthroplasty by indication. The unrevised fraction of patients with osteoarthritis is shown in green, patients receiving treatment for proximal humerus fractures in blue and patients receiving rotator cuff-related arthroplasty in red.

**Figure 2:**
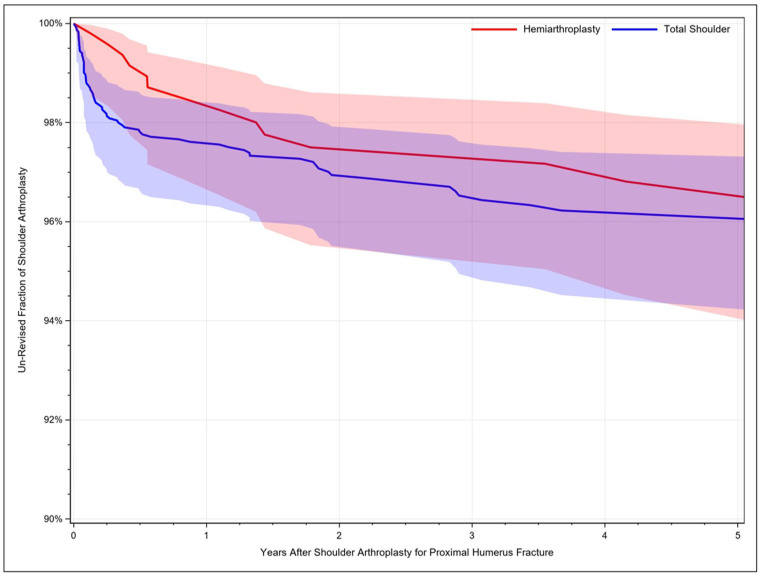
Revision rates after shoulder arthroplasty for proximal humerus fracture. The unrevised fraction of patients undergoing hemiarthroplasty is shown in red, and the unrevised fraction of patients undergoing total shoulder arthroplasty is illustrated in blue.

Between the considered time period from 2009 through 2019, a total number of 47,979 PHFs were identified. Out of these, n= 42,029 (87.6%. 95%CI: 86.6–88.5) were managed conservatively, whereas n= 3311 (6.9%, 95%CI: 6.2–7.7) received surgical treatment. A shoulder arthroplasty procedure was performed in n = 2639 (5.5%, 95%CI: 4.8–6.1) of the cases. To compare the revision rates, n = 14,775 patients with osteoarthritis and n = 4268 patients with rotator cuff arthropathy, who received a shoulder arthroplasty, served as a control group. Here, 98.1% (95%CI: 97.6–98.4) of patients with osteoarthritis, 97.1% (95%CI: 96.0–97.8) of patients with rotator cuff-related arthroplasty and 96.9% (95%CI: 95.3–98.0) of patients receiving a joint replacement for proximal humerus fracture treatment remained unrevised (Figure 1). Patients receiving a rotator cuff-related arthroplasty were more likely to require a revision compared to patients treated for osteoarthritis (HR: 1.27, 95%CI: 1.04–1.44, *p* = 0.018) and patients undergoing a shoulder arthroplasty for other reasons had a higher revision risk compared to patients with osteoarthritis, PHF and rotator cuff-related arthroplasty (Table 1). Concerning PHF management, one year after performed hemiarthroplasty (HA), 98.2% (95%CI: 96.5–99.1) of the cases remained unrevised, whereas 97.6% (95%CI: 96.3–98.4) were complication free after TSA. The five-year survivorship of the implant was 96.3 (95%CI: 93.8–97.9) after HA and 96.1% (05%CI: 94.2–97.3) after TSA (Figure 2). Identified significant risk factors for revision surgery after shoulder arthroplasty independent of the indication included age ≤ 75 years, male sex, and osteoporosis (Table 2).

## 4. Discussion

The purpose of this study was threefold, (1) to compare revision rates after osteoarthritis-related, PHF-related, and rotator cuff-related shoulder arthroplasty, (2) to compare revision rates after hemiarthroplasty and total shoulder arthroplasty for the management of PHF, and (3) to analyze risk factors for revision surgeries.

The comparison of revision rates for patients with different indications revealed that patients receiving a rotator cuff-related arthroplasty were more likely to require a revision compared to patients treated for osteoarthritis (HR: 1.27, 95%CI: 1.04–1.44, *p* = 0.018) and patients undergoing a shoulder arthroplasty for other reasons had a higher revision risk compared to patients with osteoarthritis, PHF and rotator cuff-related arthroplasty. Identified significant risk factors for revision surgery after shoulder arthroplasty included age ≤ 75 years, male sex, and osteoporosis independent of the underlying indication. In line with these findings, multivariable analysis of data from the Mayo Clinic Total Joint Registry suggested that male sex (HR compared to female sex: 1.72, 95%CI:1.28–2.31, *p* < 0.01) and rotator cuff disease (HR compared to rheumatoid arthritis: 3.99, 95%CI:1.91–8.36, *p* < 0.01) are independent risk factors for revision surgery [23]. Although not significant, our data revealed a higher risk for revision after TSA for proximal humerus fractures compared to osteoarthritis (HR: 1.19, 95% CI: 0.87–1.63, *p* = 0.272). This may have been influenced by the fact that PHFs are often surgically managed by trauma surgeons, whereas elective surgeries are mainly performed by orthopedic surgeons specializing in shoulder surgery. However, notably, another study also identified the diagnosis of osteoarthritis as an individual risk factor for the second revision of shoulder arthroplasty [24]. According to a recent meta-analysis, the most common indications for revision surgery were loosening (20%, 601/3041), instability (19%, 577/3041), rotator cuff failure (17%, 528/3041), and infection (16%, 490/3041) [19], whereas other studies reported instability to be the most frequent reason [25,26]. Whereas the BMI could not be included in our multivariate risk analysis, it was reported that persistent instability was more common in those patients with a BMI greater than 35 kg/m^2^ (hazard ratio [HR], 5; 95% CI, 2–16; *p* = 0.008) [27]. Further, it was shown that dislocation was increased one year postoperatively after primary TSA or RSA in patients who were superobese compared with patients who were not obese (2.9% vs. 1.7%, *p* < 0.05) [28]. Thus, the BMI as an individual risk factor should be considered when planning the size of glenospheres and the lateral offset at the time of RSA [27].

Further, the comparison of revision rates after hemiarthroplasty and total shoulder arthroplasty for the management of PHF revealed no statistically significant difference. Both procedures were associated with high long-term implant survivorship of 96.3 (95%CI: 93.8–97.9) and 96.1% (05%CI: 94.2–97.3), respectively, after 5 years. Earlier work utilizing data from 1993 to 2013 suggested a higher revision rate for HAs (12.7%) compared to TSA (6.7%) and RSA (3.9%), which could not be confirmed by our analysis [29]. In addition, in the Norwegian Arthroplasty Register, five-year failure rates of hemiprostheses were lower at 6% (95% CI: 5–7) compared to reversed total shoulder replacements with 10% (95% CI: 5–15) [30]. In addition, the unrevised percentage of cases was slightly higher than reported in previous studies, determining implant survival rates following TSA of 94.2% (93.2–95.3%) after 5 years, 90.2% (88.7–91.7%) after 10 years, and 81.4% (78.4–84.5%) after 20 years for the time period 1976 to 2008. The difference might be attributable to advances in implant development, although it was found that adjusted clinical outcomes (*p* = 0.048), but not the revision rates (*p* = 0.3), were significantly better for articles reporting more recent TSA procedures published between 1990 to 2015 [31]. More recent studies demonstrated results similar to ours with implant survival rates of 98.7%, 97.5% and 95.3% at two, 5 and 10 years after primary RSA, respectively [32]. For consecutive TSA performed for osteoarthritis, the 5-year implant survival estimate was 98.9% (95% confidence interval: 97.3–100%) in patients aged older than 70 years, suggesting that age alone should not be considered an indication for RSA over TSA [5]. Likewise, Gill and colleagues also reported the absence of survivorship differences at 4 years, comparing revision rates of reverse total shoulder arthroplasty (3.0%, 95% CI 2.6–3.5) and anatomic shoulder arthroplasty (3.5%, 95% CI 2.9–4.2) for the treatment of osteoarthritis in large registry data [33]. Similarly, here, no preference for choosing between HA and TSA in elderly patients can be suggested based on the presented data.

### Limitations

The Medicare data used in this study is fundamentally a set of administrative claims records, with all the attendant limitations for this type of data for orthopedic outcomes research [34,35]. First, the cohort only includes patients aged 65 or older, which has to be considered for the generalizability of the results, especially regarding osteoarthritis-related and rotator cuff-related arthroplasty, which can also occur in younger patients. Further, we do not have access to the underlying clinical records of these patients. Thus, many important clinical measures (e.g., radiologic imaging findings) and other indicators (e.g., blood chemistry, organ function test), as well as implant design, are not captured by the diagnosis or procedure codes in these data. Whereas an individual record could include errors of omission or commission, systematic error permeating throughout millions of such records in the entire Medicare system is not likely. In this regard, the broad range of facilities submitting these claims and the tens of millions of such records processed by Medicare is its unique strength. It can be assumed that the extensive information on patient characteristics and complications has a high level of quality due to its relevance for the billing of costs and the insertion of the data by appropriate specialists. In terms of the range of available parameters, the Medicare dataset is characterized by a richness of relevant parameters that is incomparable to other registry data. In addition, as PHFs included in the analysis were identified based on ICD-9 and ICD-10 codes, no subgroup analyses according to a classification of the fractures (e.g., AO, Neer) were feasible. In addition, the available CPT codes did not allow for more detailed discrimination of treatment modalities (e.g., discrimination between anatomical and reverse total shoulder arthroplasty), which would have been very desirable from the authors’ point of view. A more detailed coding describing clinical reality should be implemented in the future to ease and improve the relevance and impact of studies similar to the present.

## 5. Conclusions

In conclusion, high implant survival was found for hemiarthroplasty and total shoulder arthroplasty for the treatment of proximal humerus fractures in elderly patients. The risk of revision surgery was elevated in patients receiving a rotator cuff-related arthroplasty as well as in patients with osteoporosis, male patients and patients older than 75 years.

## Figures and Tables

**Table 1:** Comparison of risk for a revision after shoulder arthroplasty between patients treated for osteoarthritis, proximal humerus fracture and patients receiving rotator cuff-related arthroplasty. HR = hazard ratio.

Indication	Reference Indication	HR	Lower HR	Upper HR	*p*-Value
Osteoarthritis	Other Reasons	0.47	0.30	0.72	<0.001
	Proximal Humerus Fracture	0.84	0.61	1.15	0.272
	Rotator Cuff Arthropathy	0.79	0.65	0.96	0.018
Other Reasons	Osteoarthritis	2.13	1.38	3.30	<0.001
	Proximal Humerus Fracture	1.79	1.07	2.99	0.027
	Rotator Cuff Arthropathy	1.68	1.08	2.64	0.023
Proximal Humerus Fracture	Osteoarthritis	1.19	0.87	1.63	0.272
	Other Reasons	0.56	0.33	0.94	0.027
	Rotator Cuff Arthropathy	0.94	0.67	1.32	0.737
Rotator Cuff Arthropathy	Osteoarthritis	1.27	1.04	1.54	0.018
	Other Reasons	0.59	0.38	0.93	0.023
	Proximal Humerus Fracture	1.06	0.76	1.48	0.737

**Table 2:** Multivariate analysis of risk factors for revision surgery after shoulder arthroplasty. HR = hazard ratio.

Factor	HR	Lower HR	Upper HR	Chi-Sq	*p*-Value
Age 70–74 vs. 65–69 years	0.94	0.77	1.15	0.36	0.546
Age 75–79 vs. 65–69 years	0.66	0.52	0.83	12.21	<0.001
Age 80+ vs. 65–69 years	0.41	0.30	0.56	32.87	<0.001
Female sex	0.75	0.62	0.90	9.37	0.002
Anticoagulant Use	0.93	0.60	1.43	0.11	0.737
Chronic Obstructive Pulmonary Disease	1.08	0.83	1.39	0.32	0.569
Cerebrovascular Disease	1.08	0.80	1.47	0.25	0.617
Chronic Kidney Disease	1.18	0.88	1.58	1.26	0.262
Congestive Heart Failure	1.30	0.92	1.84	2.22	0.136
Diabetic	0.86	0.69	1.06	2.05	0.152
Fall Related Fracture	1.28	0.55	2.99	0.33	0.565
Hypertensive Disease	1.05	0.87	1.27	0.27	0.603
Ischemic Heart Disease	1.00	0.80	1.26	0.00	0.971
Morbid Obesity	1.30	0.85	2.01	1.43	0.231
Osteoporosis	1.38	1.02	1.86	4.47	0.035
Rheumatoid Disease	1.37	0.97	1.94	3.26	0.071
Tobacco Dependence	1.11	0.65	1.91	0.16	0.694
% College Degree	1.00	0.98	1.02	0.07	0.787
% Poverty	0.99	0.95	1.03	0.22	0.636
% Unemployed	1.01	0.93	1.11	0.09	0.766
County Population	1.00	0.99	1.01	0.14	0.708
Median HH Income	0.98	0.92	1.05	0.22	0.639

## Data Availability

The data that support the findings of this study are available on request from the corresponding author.

## Appendix A

**Table A1:** Clinical indicator used.

Condition	ICD-9	ICD-10
Osteoporosis	733.0.x	M80.x, M81.x
Osteoporotic Fracture	733.1.x	M80.x, M8.4 x
Diabetes mellitus	250.x	E08.x, E09.x, E10.x, E11.x, E13.x
Morbid Obesity	278.01, V85.4.x	E66.01.x, E66.2.x, Z68.4.x
Rheumatoid Disease	714.0.x	M05.4.x, M05.5.x, M05.7.x, M05.8.x, M05.9.x, M06.0.x, M06.2.x, M06.3.x, M06.8.x, M06.9.x
Chronic Kidney Disease	585.x	N18.x
Tobacco Dependency	305.1.x	F17.20.x, F17.21.x, F17.22.x, F17.29.x
Anticoagulant Use	V58.61.x	Z79.01.x
Opioid Use	304.0.x, 304.7.x, 305.5.x	F11.1.x, F11.2.x, F11.9.x
NSAID Use	V586.4.x	Z79.1.x
Hypertension	401.x, 402.x, 403.x, 404.x, 405.x	I11.x, I12.x, I13.x, I14.x, I15.x
Ischemic Heart Disease	410.x, 411.x, 412.x, 413,x, 414.x	I21.x, I22.x, I23.x, I24.x, I25.x
Cerebrovascular Disease	430.x, 431.x, 432.x, 433.x, 434.x, 435.x, 436.x, 437.x, 438.x	I60.x, I61.x, I62.x, I63.x, I64.x, I65.x, I66.x, I67 x, I68.x, I69.x
Chronic Obstructive Pulmonary Disease	491.2.x, 493.2.x, 496.x, 518.8.x	J44.x
Congestive Heart Disease	428.0.x, 428.1.x, 428.2.x, 428.3.x, 428.4.x, 428.9.x	I50.x
Fall Related	E88.x, E98.7.x	W0.x, W1.x
Vehicle Crash Related	E81.x, E820.x, E821.x, E822 x, E823.x, E824.x, E825.x	V0.x, V1.x, V2.x, V3.x, V4.x, V5.x, V6.x, V7.x
